# Activation of Toll-Like Receptors 2 by High-Mobility Group Box 1 in Monocytes from Patients with Ischemic Stroke

**DOI:** 10.7508/ibj.2016.04.006

**Published:** 2016

**Authors:** Leila Sadat-Hatamnezhad, Asghar Tanomand, Javad Mahmoudi, Siamak Sandoghchian Shotorbani

**Affiliations:** 1 Department of Dermatology, Tabriz University of Medical Sciences, Tabriz, Iran; 2 Department of Microbiology, Maraghe University of Medical Sciences, Maraghe, Iran; 3 Neuroscience Research Center, Tabriz University of Medical Sciences, Tabriz, Iran; 4 Department of Immunology, Tabriz Branch, Islamic Azad University, Tabriz, Iran

**Keywords:** Toll-like receptor 2, High-mobility group box 1, Stroke

## Abstract

**Background:**

Stroke is a leading cause of death all around the world, and ischemic stroke is considered to be the most common stroke type. Toll-like receptors (TLRs) are important molecules for detection of both pathogen invasion and tissue damage. In this regard, the purpose of this study was to assess the expression level of TLR2 on monocytes in patients with ischemic stroke and to evaluate the expression change profile following high-mobility group box 1 (HMGB1) stimulation.

**Methods:**

A total of 30 patients with ischemic stroke were enrolled from November 2013 to September 2014. The real-time PCR and ELISA assays were applied to detect the concentrations of TLR2 mRNAs.

**Results:**

TLR2 expression was found to be increased in patients with ischemic stroke, as compared to the healthy control group (*P*<0.001). Also, anti-TLR2 antibodies were able to decrease the expression levels of IL-17, IL-6 and IL-33. This result implies that the enhanced TLR2 pathway and Th17 cell polarization may be due to HMGB1 stimulation in ischemic stroke.

**Conclusion:**

Further clinical studies are needed for development of a new treatment strategy to inhibit the HMGB1 pathway, thus preventing the inflammation in ischemic stroke patients.

## INTRODUCTION

Stroke is a major cause of morbidity and mortality worldwide[1]. In point of fact, ischemic stroke results from loss of blood supply to a part of the brain, initiating the ischemic cascade[2]. Several studies have demonstrated the relationship between stroke and innate immune responses, which can be mediated by receptors such alike Toll-like receptors (TLRs)[3-5]. TLRs, which is a family of pattern recognition receptors, play key roles both in the activation of immune responses and in the pathogenesis of inflammatory diseases[6]. Currently, 15 TLRs have been identified in human beings, 10 of which are functional[7].

TLR2, a member of the TLR family, is a conserved pattern recognition receptor expressed on monocytes. TLR2 has the ability to detect motifs of pathogens and induce innate and adaptive immunity[8]. Therefore, TLRs may be essential receptors to detect a high-mobility group box 1 (HMGB1) during inflammation[9]. TLR2 deficiency leads to increased Th17 infiltrations in experimental brain abscesses[10]. HMGB1 is a damage-associated nuclear protein that can be secreted or released from immune, dead and dying cells. A variety of studies have shown that HMGB1 creates a positive feedback in the secretion of some pro-inflammatory cytokines from monocytes[11-13]. HMGB1 is released actively from macrophages and monocytes stimulated by lipopolysaccharide or tumor necrosis factor-alpha and passively from damaged cells and necrotic cells. Extracellular HMGB1 signals through a specialized receptor for advanced glycation end products, TLR2 and TLR4[12]. It has been reported that HMGB1 exerts multiple functions depending on its cellular localization[12]. In middle cerebral artery occlusion models in rodents, Chopp and coworkers[14] have been demonstrated that HMGB1 levels are immediately decreased in the ischemic core and in turn, serum HMGB1 levels are rapidly increased.

The aim of this study was to assess the effects of HMGB1 on the TLR2 pathway and Th17 cell infiltration in patients with ischemic stroke. For this purpose, we sought to evaluate the expression level of TLR2 on monocytes in patients with ischemic stroke. We also measured the expression change profile of TLR2 in response to *in vitro* HMGB1 stimulation to show the role of TLR2 in monocyte activation in stroke patients. In addition, we examined IL-17, IL-33 and IL-6 plasma levels, which were produced by HMGB1-stimulated monocytes and obtained from the patients.

## MATERIALS AND METHODS

### Reagents

TLR2 primer was obtained from Biogen Company, Shanghai, China. Recombinant human IL17, IL-6 and IL-33 ELISA kits were purchased from Bender Med System, Vienna, Austria. HMGB1 was obtained from Invitrogen Company (USA).

### Inclusion and exclusion criteria for stroke patients

Inclusion criteria included diagnosis of ischemic stroke causing measurable neurologic deficit, onset of symptoms <3 hours before the beginning of treatment. The exclusion criteria were head trauma or prior stroke in previous three months, symptoms suggesting subarachnoid hemorrhage, arterial puncture at non-compressible site in previous seven days, history of previous intracranial hemorrhage, elevated blood pressure (systolic >185 mm Hg or diastolic >110 mm Hg), evidence of active bleeding on examination, acute bleeding diathesis, including but not limited to platelet count <100,000/mm’, heparin received within 48 hours, resulting in a partial thromboplastin time upper limit of normal, current use of anticoagulant with protrombin time >15 seconds and blood glucose concentration <50 mg/dL (2.7 mmol/L). A control group composed of non-ischemic stroke like hemorrhagic ones or other brain injuries irrelevant to strock was considered.

### Patients and real-time PCR

A total of 30 patients with ischemic stroke were enrolled from November 2013 to September 2014. The age of the patients (18 women and 12 men) ranged from 50 to 80 years old. Healthy volunteers (n=10) were chosen as a control group. Whole blood was collected from healthy volunteers and patients with ischemic stroke and drawn directly into EDTA-containing tubes. Afterwards, the blood samples were centrifuged at 4°C for 5 minutes, the supernatant was collected for cytokine measurement, and peripheral blood mononuclear cells were recovered from the pellet by standard Ficoll-Hypaque (Pharmacia, Uppsala, Sweden) density centrifugation[15]. Monocytes were isolated from peripheral blood mononuclear cells, cultured at a density of 1×10^6^ cells/ml in RPMI-1640 medium (Gibco, Carlsbad, CA, USA) and stimulated with 0.5 µg/ml HMGB1 for 1, 2, 4, 8 and 12 hours. To block TLR2 or HMGB1, the cells were pre-incubated with 10 µg/ml rabbit anti-human TLR2 (Abcam, Cambridge, UK) for 1 hour before HMGB1 treatment. The cell suspensions without the addition of HMGB1 were used as a control group. Also, “relative quantification of gene expression” was carried out using RT-PCR. Quantitative real-time PCR was applied to measure the expression levels of TLR2. It should be noted that all samples were calibrated by β-actin. Total RNA was extracted from monocytes using the TRIzol (Invitrogen, USA) isolation solution according to the manufacturer’s instructions. The TLR2 mRNA levels were quantified by qRT-PCR amplification using a 7500 fast real-time PCR system (Applied Biosystem, Foster, CA, USA). The following sequences of primers were used, and RT-PCRs were carried out in triplicates.

TLR2 forward primer: 5’ caaacttgccttcccgtgagga-3’

TLR2 reverse primer: 5’-ccggttaagtccccttgagga-3’

β-actin forward primer: 5’-tgtggcaaagaatcctgac-3’

β-actin reverse primer: 5’-tgctcagtaaaacgcaccg-3’

### ELISA assay

IL-17 levels in monocyte cell culture supernatants and in the control groups were determined by a IL-17 ELISA Kit (R&D system, USA). IL-6 and IL-33 levels were measured by ELISA kits (R&D system, USA). The detection threshold of the assay was found to be 1 ng/ml.

### Statistical analysis

Data were summarized as mean±SD. The statistical analyses of the results were performed by the independent-sample *t*-test. Values for *P*<0.05 were considered to be statistically significant.

## RESULTS

### Increased TLR2 expression in monocytes

**Fig. 1:**
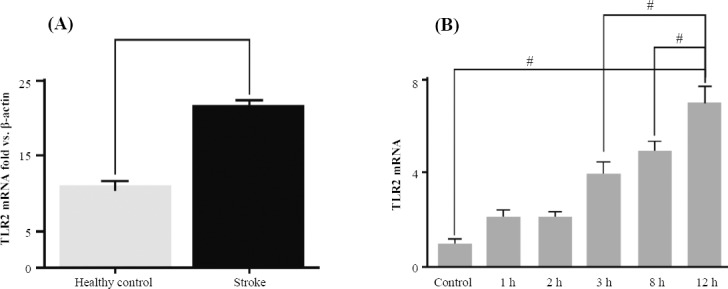
TLR2 expression results using real-time PCR. A) The real-time PCR of TLR2 expression in stroke patients and healthy controls. The Figure shows the high expression level of TLR2 in stroke patients, as compared to the controls. B) The TLR2 expression level in the HMGB-stimulated monocytes from stroke patients in a time-course manner. The TLR2 expression at 12 hours after HMGB1 stimulation is higher than that at other time courses. *#* shows *P*<0.001.

Quantitative real-time PCR results showed that the expression level of TLR2 mRNA in monocytes from patients with ischemic stroke was significantly higher than healthy volunteers (Fig. 1A). In addition, the TLR2 mRNA levels were increased in monocytes from patients when co-cultured with HMGB1, particularly after 12 hours (Fig. 1B).

### Increased cytokine concentrations in culture supernatant of monocytes stimulated by HMGB1

**Fig. 2:**
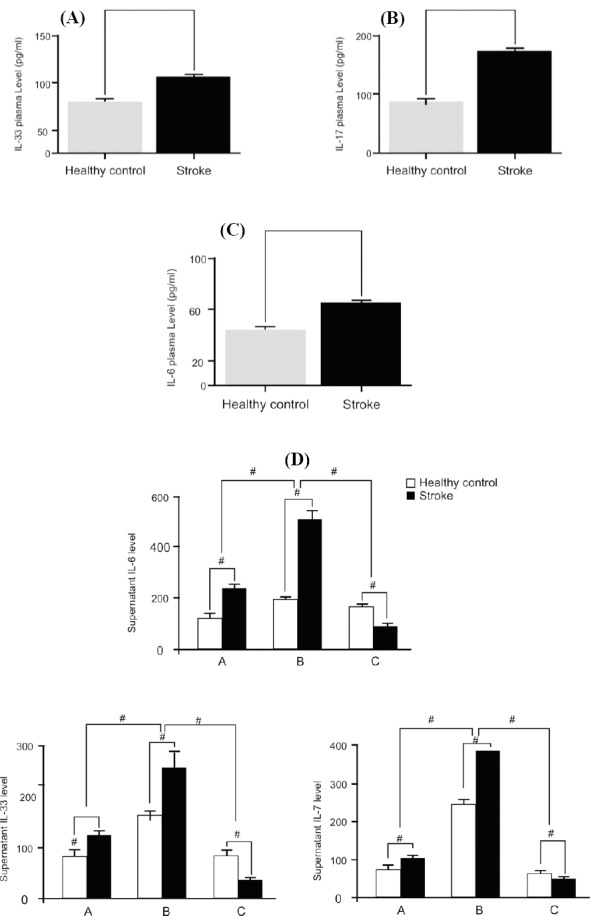
Pro-inflammatory cytokines levels. IL-33 (A), IL-17 (B) and IL-6 (C) levels in the plasma of the patients are higher than that in the healthy control. B) The IL-17 level is higher in the patient’s plasma, when compared to the healthy control. C) The IL-6 level is higher in the patient’s plasma, as compared with the healthy control. D) Cytokines levels from stroke patients and healthy controls on monocyte cells compared with HMGB1 stimulation. Part A represents the monocytes from stroke patients and healthy controls. Part B indicates the HMGB-stimulated monocytes from stroke patients and healthy controls, cultured with HMGB1 at 12 hours. Part C shows the application of anti-TLR2 antibody in the co-culture system. *#* shows *P*<0.001.

As illustrated in Figure 2A, IL-33 plasma levels were obviously increased in the ischemic stroke patients (84±4.23 pg/ml) when compared to the healthy volunteers (36.25±11.8 pg/ml). Additionally, the plasma concentrations of IL-17 in patients with ischemic stroke (165.8±17.8) were significantly higher than those in healthy volunteers (89±5.4 pg/ml) (Fig. 2B). Figure 2C shows the results of IL-6 assey in plasma from patients and healthy volunteers, representing that the IL-6 level in ischemic stroke patients is higher than healthy control subjects. In co-culture with HMGB1, the cytokine assay showed that the IL-17, IL-6 and IL-33 levels were significantly elevated in both groups, when stimulated by HMGB1 after a 12-hour incubation (Fig. 2D). To demonstrate the effect of HMGB1, monocytes were pre-treated with an anti-TLR2 antibody (10 ng/ml) for 1 hour before treatment with HMGB1, and the cell supernatant was then collected for ELISA. The data showed that the anti-TLR2 antibody is able to decrease the expression of IL-17, IL-6 and IL-33, and IL-17 and IL-33 are highly increased in stroke patients.

## DISCUSSION

An injured tissue has the ability to induce inflammatory responses due to the release of endogenous mediators, which leads to the activation of signaling cascades and, in turn, promotes T-cell expansion[15]. Some studies have demonstrated[15-17] that injured tissues are able to mediate their effects by signaling through TLR4. However, TLR2 and TLR4 are independently associated with poor outcome and correlated with higher serum levels of IL-1β, IL-6 and tumor necrosis factor α. TLR4 is also independently related to lesion volume[7,16].

TLR2 signaling is stimulated in response to host-derived molecules, including gp96 HMGB1, and possibly to larger hydrophobic molecular complexes (Hyppo’s)[17]. Investigations have shown that a variety of endogenous ligands, such as HMGB1, are expressed by damaged brain cells[16,17]. In a study, it has been revealed that dying glioma cells release HMGB, thus stimulating TLR2-dependent NFkB signaling and denderetic cells activation[18]. Another study has demonstrated that high mobility group box1-specific antibody had little effect on suppressing inflammatory cytokine expression on the first day after stroke onset compared with the Prx- specific antibodies, which can be explained by the fact that the extracellular release of HMGB1 is mostly diminished in the ischemic brain within 6 h after stroke onset[19].

Brea and coworkers[20] have analyzed the TLR2 and TLR4 expression at a protein level by flowcytometry in blood samples from patients with ischemic stroke (at 24-h, 72-h and 7-day time points), as well as in healthy controls subjects. Comparing with these studies, we sought to describe the effect of HMGB1 on TLR2 expression in monocytes in a time-course manner.

The results of the present study indicated that TLR2 mRNA was expressed at high levels in monocytes, when compared with β-actin. However, an increase in both TLR2 and β-actin mRNA expression showed that the TLR2 expression was increased in patients with ischemic stroke, as compared to the healthy control group (*P*<0.001). HMGB1, a universal nuclear protein, can mediate inflammation and plays an important role in the pathogenesis of various inflammatory diseases. However, it can be deduced that the immune system in stroke patients is in part regulated by an increased frequency of Th17 cells, which leads to increases in TLR2 and inflammatory cytokines. In addition, the elevated levels of IL-17, IL-33 and IL-6 were found in the plasma of patients with ischemic stroke, as well as in the supernatants of the cell monocyte stimulated by HMGB1. Interestingly, the enhanced expression of TLR2 on monocytes was also detected in stroke patients compared with healthy volunteers.

From the results of this study it can be concluded that HMGB1 is able to mediate endogenous TLR2 activation, and enhanced TLR2 levels may be due to HMGB1 stimulation.
